# Modulation of Morpho-Physiological and Metabolic Profiles of Lettuce Subjected to Salt Stress and Treated with Two Vegetal-Derived Biostimulants

**DOI:** 10.3390/plants12040709

**Published:** 2023-02-06

**Authors:** Francesco Cristofano, Christophe El-Nakhel, Giuseppe Colla, Mariateresa Cardarelli, Youry Pii, Luigi Lucini, Youssef Rouphael

**Affiliations:** 1 Department of Agricultural Sciences, University of Naples Federico II, Via Università 100, 80055 Portici, Italy; 2 Department of Agriculture and Forest Sciences, University of Tuscia, 01100 Viterbo, Italy; 3 Faculty of Science and Technology, Free University of Bozen/Bolzano, 39100 Bolzano, Italy; 4 Department for Sustainable Food Process, Research Centre for Nutrigenomics and Proteomics, Catholic University of the Sacred Heart, 29122 Piacenza, Italy

**Keywords:** plants extracts, *Lactuca sativa* L., polyphenols, flavonoids, anthocyanins, NaCl, yield, ROS

## Abstract

Salinity in water and soil is a critical issue for food production. Using biostimulants provides an effective strategy to protect crops from salinity-derived yield losses. The research supports the effectiveness of protein hydrolysate (PH) biostimulants based on their source material. A greenhouse experiment was performed on lettuce plants under control (0 mM NaCl) and high salinity conditions (30 mM NaCl) using the Trainer (T) and Vegamin (V) PH biostimulants. The recorded data included yield parameters, mineral contents, auxiliary pigments, and polyphenolics. The plant sample material was further analyzed to uncover the unique metabolomic trace of the two biostimulants. The results showed an increased yield (8.9/4.6%, T/V) and higher photosynthetic performance (14%) compared to control and salinity treatments. Increased yield in salinity condition by T compared to V was deemed significant due to the positive modulation in stress-protecting molecules having an oxidative stress relief effect such as lutein (39.9% 0 × T vs. 30 × V), β-carotene (23.4% vs. V overall), and flavonoids (27.7% vs. V). The effects of PH biostimulants on the physio-chemical and metabolic performance of lettuce plants are formulation dependent. However, they increased plant growth under stress conditions, which can prove profitable.

## 1. Introduction

Soil and irrigation water salinity are outstanding problems threatening food security in a world with an ever-growing population. FAO estimates salt-affected soil area at more than 4.4% of total land area, with arid and semi-arid regions being the most affected [1]. Salinization is a double-faced problem in the Mediterranean region, as freshwater availability is becoming scarce due to climate change-induced droughts, and the use of saline water for irrigation can aggravate this issue, resulting in lower-than-expected yields [2]. In plants, salt stress is described as the combination of an early onset drought-like condition due to the increase in osmotic pressure at the root level and a later toxic effect of both sodium and chloride ions. Osmotic stresses damage plant tissues by determining reactive oxygen species (ROS) formation due to limited water availability, which can gravely depress photosynthetic performance [3]. Furthermore, toxic sodium ions compete for the same cationic transporters and ion channels as potassium, and this interchange can cause a further depression of the plants’ metabolic performances [4].

Farmers have many strategies to cope with yield depression due to saline conditions, such as water and nutrient management, the switch to soilless agriculture and the use of salt resistant-genotypes [2,5,6]. A further addition to the farmer toolset is represented by plant biostimulants (PBs), which are formulations proven to be particularly effective in alleviating yield losses due to suboptimal conditions [7]. Protein hydrolysate (PH) biostimulants, which are a mixture of amino acids and peptides obtained via the hydrolysis of protein matrices [8,9], can be rapidly deployed via foliar spray or substrate drench [10], and are a well-studied and well-proven category. A recent meta-analysis by Li and collaborators [7] found an average 16.5% marketable yield increase across the available PH literature, furthering their usefulness. Among this product category, vegetal-derived PHs deriving from enzymatic hydrolysis are more environmentally friendly options compared to those derived from chemical hydrolysis. This is especially true when considering the plethora of waste biomass from crop cultivation that could be used for agrochemicals production [10,11]. Research has summarized the activity of PH biostimulants as the increase in root growth to the presence of an auxin-like action, thus providing for higher nutrient uptake, the stimulation of carbon and nitrogen metabolism and the priming effect of the antioxidant systems that fend off plant stresses [9]. This effect has been postulated to come from the presence of bioactive molecules, such as signaling peptides, of which the root hair promoting peptide is the most widely reported as being found in the Trainer biostimulant [10].

One of the techniques that has furthered the understanding of these products has been metabolomics, which offers a picture of the mode of action by identifying markers of the changes in plant metabolism. For instance, in drought-stressed tomato (*Solanum lycopersicum* L.) and salt-stressed lettuce (*Lactuca sativa* L.), the metabolic profile change to the application of the Trainer biostimulant has been described as the reprogramming of the phytohormone profile, which has resulted in improved resistance to oxidative stresses [12,13]. During salt stress, this leads to the hypothesis that the increase in root absorbing area and the priming of antioxidant-related defense mechanisms can lead to decreased toxic ion absorption and better oxidative status, thus increasing plant performance, as it has been found in tomato and spinach [14,15]. However, metabolomics has also provided evidence that not all vegetal-derived biostimulants are equal in their modulation of the metabolome. A recent study by Ceccarelli and collaborators [16], which tested vegetal PH biostimulants derived from five distinct protein matrices, found an accumulation of auxins and gibberellins in tomato root tissue on two of the tested formulations and an opposite behavior in the remaining three. This led them to consider that, due to the intrinsic variation in the protein makeup of the source matrix, a generalized approach to vegetal PHs cannot be taken [16].

There is an argument to be made about the composition of these products: their nitrogen content, as a proxy of the content of the nitrogen-containing active molecules, may explain the source of the variability seen in the literature. Recent research conducted on lettuce elucidated that foliar application rates of the Trainer biostimulant as high as double the normal rate increased both photosynthetic rate and nutrient uptake (P, S, K) compared to the base treatment [17]. While this may confirm that a higher supply may also entail an increase in active molecules supplied, it has also been found on lettuce that an overuse may cause growth regression, which has been found to be cultivar specific [18].

To test the validity of PH biostimulants in ameliorating salt stress tolerance, we set out a greenhouse experiment comparing two commercially available vegetal-derived PH biostimulants that differ in their composition (crucially, in the amount of nitrogen), and potentially, active ingredients content. We also tested both their salt stress and ameliorating effects via morpho-physiological and biochemical assays to confirm that the modulation of metabolic markers varies on a product-to-product basis. For this purpose, we also selected lettuce as the test crop, as it is a prime candidate for determining the stress-ameliorating power of biostimulants due to being widely cultivated and being a glycophyte, or moderately sensitive to salinity [18,19]. This study may shed further light on PH biostimulants and may further the argument for using such products in agriculture under suboptimal conditions.

## 2. Results

### 2.1. Lettuce Growth and Morphometric Parameters

The impact of salinity and the biostimulant treatments on the growth of lettuce plants can be seen in Table 1. Overall, salinity significantly impacted all studied parameters save for the leaf number data. In particular, leaf area decreased by 14.0% and fresh weight by 23.3%, whereas leaf dry matter increased by 18.7%.

Biostimulant treatments significantly increased the shoot fresh weight when averaged across nutrient solution (NS) conditions. Data show the Trainer biostimulant being the most effective, as plants treated with this formulation recorded an 8.9% increase when compared to the untreated control, and 4.1% when compared to the Vegamin treatment; the latter also showed 4.6% higher figures compared to the control.

Leaf area was affected by the interaction between the biostimulant and the NS treatment; biostimulant-derived differences in the 0 mM NaCl group were deemed non-significant compared to the control. On the contrary, in salt conditions, both the Trainer and Vegamin treatments managed to increase this parameter by 5.3% on average.

### 2.2. Leaf Photosynthetic and Biochemical Parameters

The impact of salinity and the biostimulant treatments on the studied photosynthetic and biochemical parameters of lettuce plants can be seen in Table 2. Again, the impact of the salinity treatment was present across the board. Lower leaf stomatal conductance (15,8%), transpiration (13.9%) and thus higher intrinsic water use efficiency (18.7%) were denoted under salinity.

However, when biostimulants enter the picture, a S × B interaction was found only in the leaf CO_2_ assimilation rate. The application of the different biostimulants did not engender a significant increase in 0 mM NaCl condition, whereas both biostimulants under salinity (30 mM NaCl) increased A_CO2_ by 24.6% on average. When averaged across the NS, Vegamin application increased g_s_ by 18.8%, and Trainer application increased WUEi by 13.5% compared to the untreated control (0 mM NaCl).

All studied biochemical parameters significantly increased when lettuce plants were treated with the high-salt NS treatment (Table 2). Our results showed higher proline (134.5%), MDA (10.5%) and H_2_O_2_ (52.1%) contents under salinity stress (30 mM NaCl).

Save for the peroxide content, our results denote how the S × B interaction modulated the content of these stress markers in lettuce leaves. When proline under high salinity is considered, both biostimulants significantly decreased their contents by 24.3% on average compared to the untreated high salt control. The results also show that MDA contents were significantly lowered in both NS conditions by the studied biostimulant treatments; on average, both Trainer and Vegamin decreased this membrane oxidation parameter by 18.1% in the control condition. When salinity condition is considered, Vegamin treatment lowered MDA contents by 31.7 and 17.3% when compared to both the untreated control and the Trainer biostimulant and brought this parameter down to the 0 mM NaCl × biostimulants level.

### 2.3. Leaf Mineral Contents

The impact of salinity and the biostimulant treatments on the accumulation of leaf minerals can be seen in Table 3. Salinity condition impacted plant nutrient accumulation, as the concentration of sulfur, calcium and magnesium all decreased by 7.9, 23.1 and 13.8%, respectively. Sodium and chloride contents were understandably affected, as we recorded an increase of 271.5 and 125.7%, respectively; this is particularly evident in the Na/K ratio, which increased three-fold due to sodium accumulation.

When considering the biostimulant treatments, Trainer effectively raised calcium and magnesium contents by 19.7 and 13.8%, respectively, when averaged across the NS treatments. When looking at S × B interactions, both biostimulants managed to decrease sodium accumulation compared to the salinity control by 37.2% on average, thus decreasing the Na/K ratio by 30.4%, whereas these values were unchanged in 0 mM NaCl condition.

### 2.4. Leaf Pigment Content and Antioxidant Activity

The impact of salinity and the biostimulant treatments on the accumulation of leaf pigments and antioxidant activity can be seen in Table 4. A general across-the-board increase in β-carotene content and antioxidant activity was noted in salinity treatment.

However, when the biostimulants are considered, interaction data show that the Trainer biostimulant was the only treatment that increased lutein content by 45.1% in the salinity condition compared to the untreated control. In contrast, in the 0 mM NaCl condition, no effect could be observed. The data showed no significant differences across NS treatments between the biostimulant treatments and the untreated control in the case of β-carotene. However, a 19% difference was deemed significant between Trainer and Vegamin. In addition, β-carotene increased under salinity by 30.3%.

When averaged across NS treatments, all the considered antioxidant assay data were significantly affected by the PH treatments, and the Trainer formulation yielded the highest results in all cases. DPPH, ABTS and FRAP data showed increases by Trainer and Vegamin of 30.6, 32.5, 58,2% and 19.5, 32.5 and 29.5%, respectively, compared to the untreated control. These antioxidant assays were boosted by salinity stress (30 mM NaCl) by 9.31, 11.5 and 12.2% for DPPH, ABTS and FRAP, respectively.

### 2.5. Leaf Polyphenolic Contents

The impact of salinity and the biostimulant treatments on the modulation of leaf polyphenolic contents can be seen in Table 5 and Table 6. The salinity treatment increased the concentration of the assayed phenolic acids and flavonoids.

Chlorogenic acid was largely the most represented phenolic acids, accounting for 93.9% of the total phenolic acids content when both nutrient solution conditions are averaged, and its concentration increased 12.6% in salt stress. Save for ferulic acid, which was unaffected by all treatments, all the studied phenolic acid compounds accumulated in response to biostimulant applications. Trainer and Vegamin increased chlorogenic acid concentration by 39.1% when averaged across NS treatments, and this increase was largely responsible for the differences in the total amount of phenolic acids. The second most abundant components among the assayed phenolic acids were the sinapic acid conjugates (represented as synapoyl-hexose), which also showed an S × B interaction. In salinity condition, the 30 mM NaCl × Trainer treatment showed the highest concentration of these compounds when compared to both Vegamin (24.5%) and the untreated control (71.4%). In comparison, in the 0 mM NaCl condition, both biostimulants engendered no significant effects when compared to the control. A similar trend was shown regarding coumaric acid esters (represented as coumaroyl-diglucoside) and disinapoylgentobiose, of which the Trainer-treated plants accumulated the most across NS treatments, +69.6 and +50.0% when compared to the untreated control, respectively.

Flavonoids data showed very similar outcomes. When the S × B interaction data of the total flavonoids content are considered, Trainer induced the highest accumulation in both low-salt (136.2% and 33.1%) and salt conditions (67.0% and +3.0%) compared to both the untreated control and Vegamin, respectively. The principal driver of the flavonoid profile was quercetin-3-glucoside, which accumulated the most in the leaves of Trainer-treated plants. Trainer induced a 2.46-fold accumulation in control conditions compared to the untread plants, and a 69.2% increase in stress conditions. When Vegamin is considered, the increases were 77.0% and 35.3%, respectively. Both isorhamnetin 3-rutinoside and kaempferol 3-glucoside showed S × B interactions; the data showed that biostimulants increased the amount of these compounds in both stress and non-stress conditions. The formulations yielded similarly higher concentrations in control conditions; however, Trainer elicited the highest accumulation in high salinity levels (59.5 vs. 33.3%, and 53.2 vs. 30.2%, Trainer vs. Vegamin, respectively, compared to the untreated control). When averaged across NS treatments, Kaempferol 3,7-diglucoside was down-accumulated by 32.7%, on average in biostimulant-treated plants, with no significant differences among the biostimulants. Lastly, strong, 4,3- and 1.3-fold increases in rutin concentration were noted as a mean effect of biostimulant and salinity treatments, respectively.

### 2.6. Principal Component Analysis

**Figure 1:**
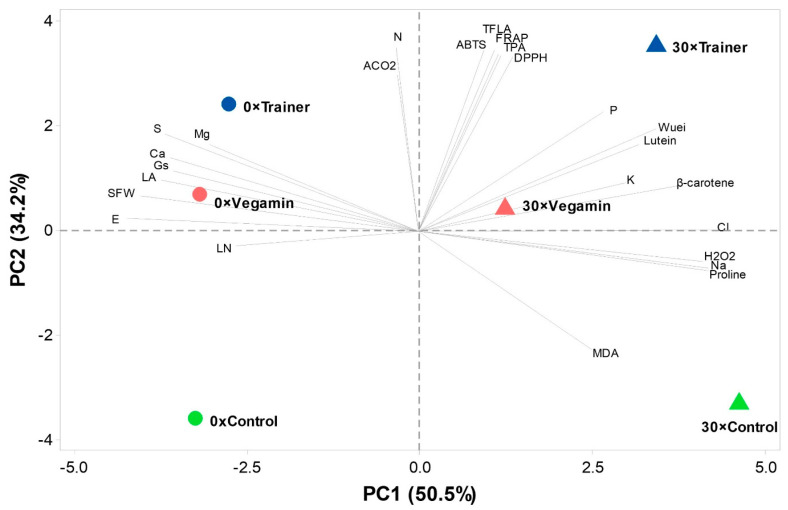
Principal component loading plot and scores of the principal component analysis (PCA) on biometric (leaf number (LN), leaf area (LA), shoot fresh weight (SFW)) physiological and biochemical (carbon dioxide accumulation (A_CO2_), stomatal conductance (g_s_), transpiration (E), proline, MDA and H_2_O_2_), mineral content (N, P, K, S, Ca, Mg, Na, Cl), auxiliary pigments (β-carotene and lutein), antioxidant activity (DPPH, ABTS, FRAP), total phenolic acids (TPA) and flavonoid contents (TFLA) of lettuce plants as modulated by salt (0, 30 mM) and biostimulant (“Trainer”, “Vegamin”) applications.

To provide a summary of the changes in the morphological, physiological and metabolic traces left by the application of both the salt stress and PHs, a principal component analysis (PCA) was carried out, which separated the treatments based on the traits associated with them. The principal components (PCs) 1 and 2 (Figure 1) explained 84.7% of the total variance and were both associated with eigenvalues higher than 1. PC1 explained 50.5% of the total variance and was positively correlated with higher phosphorous, potassium and sodium chloride contents, salt-stress markers such as H_2_O_2_, MDA and proline, and auxiliary pigments such as β-carotene and lutein. PC1 was negatively correlated with shoot fresh weight (SFW), leaf area, photosynthetic parameters such as stomatal conductance (gs), transpiration (E), and sulfur, magnesium and calcium contents. The second principal component (PC2) explained 34.2% of the variance and was correlated with higher CO_2_ assimilation (A_CO2_), leaf nitrogen content and antioxidative markers including ABTS, FRAP, DPPH, and total flavonoids (TFLA), while being negatively correlated with the membrane oxidation parameter MDA. As it is visible from the PCA biplot, there is separation from the studied treatments based on both salt and biostimulant combination. In particular, the left quadrant shows the presence of both Trainer and Vegamin treatments in control conditions. These treatments were associated with higher fresh weight, sulfur calcium and magnesium contents. The upper right quadrant shows both biostimulant treatments in salt-stress conditions. In particular, the 30 × Trainer treatment was associated with the increase in antioxidant markers, auxiliary pigments and phosphorous and potassium contents. Both the control treatments sit opposite from their respective biostimulant counterparts, in the lower left and lower right quadrants.

## 3. Discussion

This work aimed to test the performance of two vegetal-based PH biostimulants to provide an understanding of how they can manage to improve growth performance in salt-stressed lettuce plants. It also aimed to delineate their dissimilarities using morpho-physiological, biochemical measurements and metabolomics. We found the recorded growth increases across nutrient solution conditions to align with PH literature on leafy vegetables under stress (salt, low nutrient availability) and non-stress conditions [10]. In more practical terms, the 8.9 and 4.6% average increase in fresh weight recorded by the Trainer and Vegamin biostimulants in this work can be quantified in 3.3 and 1.7 T ha^−1^ of marketable lettuce biomass, which could prove to be economically advantageous.

However, as lettuce leaf area is a proxy for the whole plant growth, we found no difference between the two treatments in the control NS treatment. To explain the difference in marketable yield of the two biostimulants, we first need to preface that this study showed further confirmation that the leaf number parameter is under genotypical control and, at least for this cultivar, not steered by biostimulant effects as confirmed by previous research [18]. Furthermore, we found on average that leaf dry matter percentage, and water content in turn, remained equal across nutrient solution treatments.

Based on the results of our research, we can confirm the different mechanisms through which the used biostimulants ameliorated plant stress and boosted plant growth in control conditions, which were highlighted in previous research [10,14,20]. Firstly, we found an increased photosynthetic output in stress and non-stress conditions; this has been described as the plant metabolism stimulation by bio-effectors in the products such as signaling peptides [9]. Due to hormone-like effects, these compounds effectively prime plants to perform better in stress and non-stress conditions by impacting enzymes related to both carbon and nitrogen metabolism [9,18]. Additionally, we found an across-the-board improvement in plant nutrition and cell homeostasis parameters, exemplified by the decrease in proline content, the decrease in sodium content, which translated into lower Na^+^/K^+^ ratios, and the Trainer-specific increase in leaf calcium and magnesium. The regulation of cellular osmotic balance is a key stress-averting strategy that plants employ to fight off salt stress [21]. To adjust to the increased osmotic pressure due to media salinity, plants produce a variety of molecules such as proline and soluble sugars, deemed compatible solutes, which accumulate in tissues. In general, the combination of the production of such solutes and the accumulation of cell-wall-strengthening molecules such as lignin in salt stress results in increased plant dry weights [22,23], consistent with what has been recorded in our trial.

Biostimulants are known to induce an accumulation of proline in tissues subjected to osmotic stresses such as drought and salinity as a way to favor osmotic homeostasis [10] and fight off oxidative stresses [24]. However, our results show an opposite trend, contrasting what was previously obtained on salt-stressed lettuce treated with the Trainer biostimulant [13]. In their study, Lucini and collaborators [13] found no significant differences in the amount of proline in the leaves of biostimulant-treated plants grown in a saline environment compared to the untreated control. Effectively, our results show a better adaptation to the saline environment by biostimulant-treated plants, as exemplified by the lower Na contents and Na^+^/K^+^ ratio, thus providing the lower proline content. This could be explained by various factors, including biostimulant-mediated root growth and genotype strategies for salt resistance.

PH biostimulants have a proven ability to increase root growth through an auxin-like effect [13,16], and while root expansion was not evaluated in this trail, it is safe to assume that higher absorbing area in the lower substrate horizons could effectively decrease salt uptake, as higher concentrations tend to be in the upper layers due to evaporation/transpiration. However, this does not completely elucidate how sodium, and not chloride, was reduced in tissues of the treated plants. A further explanation may come from how plants reduce sodium accumulation in the shoot. Aside from vacuole sequestering, which would have manifested in the mineral analyses, sodium ions can be transported to the phloem and later, to the roots, by high-affinity potassium transporters (HKTs), and then expelled to the substrate via the salt overly sensitive (SOS) pathway [25]. This hypothesis is validated by numerous instances of biostimulant studies in the literature, whereby after the application of these substances, there was an increase in the expression of HKTs and sodium antiporters in the SOS pathway [10,26,27]. Lastly, we did not find a decrease in leaf potassium contents, which can be expected due to the ionic affinity of K^+^ and Na^+^ [21], and has been found in a recent study on lettuce plants subjected to salt stress [22]. This may be due to genotype-specific strategies, which may minimize the leaking of potassium and decrease sodium import from the substrate via the endodermis [25]. While there is no confirming evidence of this phenomenon occurring, a previous study on the same lettuce cultivar showed high phenotypical plasticity in stress conditions, namely high irradiance and heat [28].

A third stress-ameliorating mechanism manifested in this trial was the biostimulant-mediated induction of oxidative-stress defense mechanisms. Reactive oxygen species, or ROS, are formed during photosynthesis due to the salinity-derived water stresses, leading to increased hydrogen peroxide and the peroxidation of membrane lipids, disrupting metabolism [29]. ROS-derived damage can be averted by combining enzymatic and non-enzymatic systems, including auxiliary pigments (carotenoids, anthocyanins) and polyphenolics. Plant carotenoids such as β-carotene and lutein are plastid-bound pigments that serve as auxiliary pigments, as they absorb light energy and then transfer it to chlorophylls. They also work by scavenging ROS, dissipating excess light energy by generating heat, and they protect cellular membranes by reacting with lipid peroxidation reaction products, thus ending oxidative chain reactions [24,30]. Our results showed that the Trainer biostimulant provided the highest lutein contents in saline conditions compared to the untreated control and the Vegamin treatment. Lutein is a key element to photosystem II protection via the xanthophyll cycle [31], which suggests better photo-oxidative protection after applying the Trainer formulation.

Polyphenolics are a class of molecules that stem from the central phenylpropanoid pathway and serve as plant growth regulators and stress-response molecules [32]. Their function, among others, is to donate electrons to peroxidases for H_2_O_2_ detoxification, thus acting like antioxidants, and are involved in the mechanical strengthening tissues to enhance the resistance to water deficit [33]. When looking at the phenolic acid assays, both Trainer and Vegamin biostimulants increased chlorogenic acid, which is the most present phenolic acid in lettuce, in accordance with previous studies in the literature, which found similar increases [34,35]. However, when the leaf flavonoids are considered, we found quercetin-3-glucoside or cyanidin 3-glucoside, and anthocyanin [36] contents to be increased in both control and salt conditions. Due to their antioxidant activity, anthocyanins are particularly useful to plants in stressful conditions [37], and in this case, it furthers the case of Trainer being the most successful biostimulant in eliciting a plant-stress averting response.

The principal component analysis, other than providing a visual summary of the effect that the two products had on the studied features, is an effective tool to delineate their mode of action, as previous PH research shows [38]. In both control and salt conditions, the two tested biostimulants sit opposite their untreated counterparts, yet the Trainer biostimulant is associated with higher values, especially when photosynthetic and antioxidant activities are considered. This could be due to a variety of factors, but it could be safe to assume that they all stem from the product composition. As the Trainer biostimulant contains over double the nitrogen content, it could be inferred that some of the active ingredients may be actually more concentrated in this product, when compared to the Vegamin formulation. As further proof, recent research of the Vegamin biostimulant has shown that by splitting the product in its molecular fractions, especially in the <1 kDa molecular weight class, it is possible to increase some aspects related to their bioactivity, especially the ones related to oxidative stress defense [39]. This is in line with the theory surrounding the inner workings of PH biostimulants, which sees in the low molecular weight peptides the key to their action [10]. Furthermore, FRAP analysis conducted on the products shows stark distinction between the two in terms of the antioxidant power of the formulations, which may also suggest a higher capacity of the Trainer biostimulant in helping plants against the accumulation of ROS [40].

Overall, our results delineate a scenario of better protection against stresses provided by the Trainer biostimulant, which can compound to a better plant physiological state and thus the recorded higher shoot fresh weight.

## 4. Materials and Methods

### 4.1. Growth Conditions, Experimental Design and Plant Material

The greenhouse trial started on 22 March 2021 (DAT 1, or the day after transplant) and ended on 29 April 2021, for a total of 39 days. The experiment was carried out in an unheated greenhouse at the Department of Agricultural Sciences of the University of Naples “Federico II” (40°48′ N, 14°20′ E, 29 m.s.l.). Three true leaf stage seedlings of *Lactuca sativa* L. cv. “Maravilla De Verano Canasta” were transplanted into 1.6 L plastic pots containing a 90:10 (*v/v*) mixture of 3 mm quartz sand (Vaga, Sabbie e Ghiaie Silicee, Costa de’Nobili (PV) Italy) and perlite, respectively. The pots were arranged in a configuration consisting of four 35 × 20 cm double rows; thus, the planting density was set at 14 plants m^−2^. The double rows were set at a 50 cm distance.

The experimental design consisted of a split plot system, whereby each of the two couples of double rows was assigned to a tank that contained a base nutrient solution (NS) or a NS to which sodium chloride was supplied.

The composition of the base NS was: 8 mM nitrate, 1.5 mM phosphorus, 4 mM potassium, 4 mM calcium, 2.5 mM sulfur, 1.25 mM magnesium, 20 μM iron, 9 μM manganese, 0.3 μM copper, 1.6 μM zinc, 20 μM boron and 0.3 μM molybdenum. The base NS had an electrical conductivity of 1.6 dS m^−1^, whereas the addition of 30 mM of NaCl created the salinity NS treatment (4.4 dS m^−1^). The pH of the solutions was monitored and kept at 5.8 ± 0.2 with a portable pH meter (HI 991301, Hanna Instruments Italia S.R.L., Ronchi di Villafranca Padovana (PD), Italy).

The biostimulant (B) subfactor consisted of two biostimulant treatments and an untreated control, which were arranged inside the NS plots in a randomized complete block system with three replicates. Each B replicate was composed of five lettuce plants, for a total of 15 plants per biostimulant treatment, per NS plot.

### 4.2. Biostimulant Treatments

The PH biostimulants chosen for this trial were Vegamin and Trainer (Hello Nature Italia S.R.L., Rivoli Veronese (VR), Italy), both made from vegetal sources, and consisting of mixtures of amino acids and soluble peptides [38]. Quantitative analysis of both products, in accordance to Sorrentino and collaborators [41], show carbon and nitrogen contents to be (carbon) 17.6 and 17.2%, and (nitrogen) 5 and 2.2% for Trainer and Vegamin, respectively.

The amino-acidic content of the Trainer formulation has been described in a previous work by Paul and collaborators [12] as (g kg^−1^ product): Ala (12), Arg (19), Asp (33), Cys (4), Glu (54), Gly (13), His (8), Ile (12), Leu (24), Lys (19), Met (4), Phe (16), Pro (15), Ser (17), Thr (11), Trp (4), Tyr (13), and Val (16). Analogous analyses were performed on the Vegamin biostimulant, which yielded an aminogram comprising (g kg^−1^ product): Ala (7), Arg (10), Asp (18), Cys (1), Glu (33), Gly (6), His (4), Ile (5), Leu (8), Lys (9), Met (1), Phe (6), Pro (9), Ser (5), Thr (6), Trp (1), Tyr (3) and Val (5).

Both products were also subjected to further analysis to determine the ferric-reducing antioxidant power (FRAP) and total phenolic and flavonoid contents in accordance to Paul and collaborators’ [12] work, and were quantified as: (Trainer) 41.9 mmol Fe^2+^ g^−1^ f.w., 8.93 mg of gallic acid equivalent per gram of fresh product and 0.95 mg of quercetin equivalent per gram of fresh product, (Vegamin) 1.32 mM Fe^2+^ g^−1^ fresh products, 1.52 mg gallic acid equivalent g^−1^ fresh product and 0.23 mg quercetin equivalent g^−1^ fresh product.

Both biostimulants do not contain phytohormones, as previous research shows [41,42]. Foliar applications of the biostimulants, both at a rate of 3 mL formulation L^−1^ solution, were made using 10 L steel-bottle sprayers, which were tested for spraying volume consistency. Applications of the biostimulants were carried out in order to provide a uniform coat of the products on all leaf surfaces.

A total of five treatments were applied during the experiment, starting from the day after transplant (DAT) 10 and once per week.

### 4.3. Yield, Growth Assessment, Leaf Area Measurement and Sampling

At the end of the experiment (DAT 39), three plants per experimental unit were chosen for fresh weight measurements, which included leaf number, leaf area and shoot fresh weight, and later for dry matter analyses.

Leaf area measurements were carried out via leaf photography and later quantification using the ImageJ v1.52a software (U.S. National Institutes of Health, Bethesda, MD, USA) and expressed in cm^2^.

After the fresh weight measurements, all plant matter was dried in a forced-convection oven at 60 °C until a constant weight was reached. After the drying step, leaf dry matter percentage (DM%) was quantified as:

(1) $$\;\mathrm{DM}\%\;=\frac{\mathrm{Leaf}\;\mathrm{dry}\;\mathrm{weight}}{\mathrm{Leaf}\;\mathrm{fresh}\;\mathrm{weight}}\times 100$$

The obtained dry matter was further processed using a grinding mill (MF10.1 model, IKA-Werke GmbH & Co. KG, Staufen, Germany) for leaf mineral content determination.

A pool of four leaves from two plants per experimental replica was immediately quenched in liquid nitrogen and later stored at −80 °C to determine leaf proline and oxidative stress markers (malondialdehyde or MDA, and hydrogen peroxide or H_2_O_2_). A further set of fresh samples were stored at −20 °C and later freeze-dried using a model Alpha 1–4 lyophilizer (Martin Christ Gefriertrocknungsanlagen GmbH, Osterode am Harz, Germany), for the determination of leaf auxiliary pigments, antioxidant activity (DPPH, ABTS, FRAP), and polyphenolic contents.

### 4.4. Leaf gas Exchange and Biochemistry Parameters

Leaf gas exchange measurements were carried out on 28 April 2021 (DAT 38) on healthy, young and fully expanded leaves, using an LCi T compact photosynthesis system (ADC Bioscientific Ltd., Herts EN11 0NT, UK), equipped with a broad-leaf chamber and a programmable LED light. Photosyntetic photon flux density (PPFD) inside the chamber was set as 1000 μmol m^−2^ s^−1^ and airflow as 200 mL s^−1^; both relative humidity and CO_2_ concentration were kept at ambient levels. The data recorded included CO_2_ net assimilation rate (A_CO2_; μmol CO_2_ m^−2^ s^−1^), stomatal conductance (g_s_; mmol H_2_O m^−2^ s^−1^) and transpiration (E; mmol H_2_O m^−2^ s^−1^). A fourth derived measurement, instantaneous water use efficiency or WUEi, was calculated as:

(2) $$\;\mathrm{WUEi}=\frac{A{\mathrm{CO}}_{2}}{E}$$

Leaf proline, MDA and H_2_O_2_ measurements were carried out using analogous methods described by Kumar and collaborators in their previous research [43]. In brief, proline content was determined on 0.5 g of fresh tissue in three steps, namely a homogenization step in sulfosalicylic acid, a reaction in a mixture of 50:50 (*v/v*) acid-ninhydrin and glacial acetic acid, and final spectrophotometric determination of the toluene-extracted proline at 520 nm. Proline measurements were quantified as mM proline 100 g^−1^ fresh weight (FW).

Leaf MDA concentration was also determined on fresh tissue after homogenization in 0.1% trichloroacetic acid (TCA), centrifugation and reaction with thiobarbituric acid to form a 532 nm chromophore. The absorbance was recorded at 532 and 600 nm, and MDA concentration was calculated as the difference in absorbance values. MDA measurements are quantified as µM MDA 100 g^−1^ FW.

Lastly, hydrogen peroxide (H_2_O_2_) measurements were carried out on TCA-homogenized tissues after adding 10 mM K-phosphate buffer (pH 7.0) and 1 M potassium iodide. Absorbance was measured at 390 nm against an H_2_O_2_ standard, and the measurements were quantified as mM H_2_O_2_ 100 g^−1^ FW.

### 4.5. Leaf Total Nitrogen and Mineral Analysis

The total leaf nitrogen assay was conducted on dry leaf samples using the Kjeldahl method after mineralization with sulfuric acid and a potassium sulfate–copper sulfate catalyst, as described in previous works [44,45].

A further set of minerals, namely P, K, S, Ca, Mg, Na and Cl contents, were determined using the ICS-3000 ion chromatography system (Dionex, Sunnyvale, CA, USA) after water extraction of dry sample matter in an 80 °C heated bath for 10 min. After separation using the IonPac AS11-HC and IonPac CS12A analytical columns, the amount of minerals was quantified against analytical standards as described in previous work [18]. All leaf mineral contents are expressed as mg g^−1^ dry weight (DW).

### 4.6. Leaf Carotenoid Contents, Antioxidant Activity

Leaf lutein and β-carotene determinations assays were performed using 100 mg of lyophilized leaf matter. As described by Kyriacou and collaborators [46], a first sample extraction was performed in ethanol–0.1% BHT mixture, and a later saponification step was employed using KOH. Pigment extraction was carried out in n-hexane, which was then evaporated in a nitrogen flow. Thereafter, 1 ml of chloroform was added to the dry residue, and the mixture was separated using a Shimadzu Model LC 10 chromatographer (Shimadzu, Osaka, Japan) equipped with a reverse phase 250 × 4.6 mm, 5 μm Gemini C18 column (Phenomenex, Torrance, CA, USA) as described by Kyriacou and collaborators [47]. Carotenoid contents were quantified as mg kg^−1^ DW.

The spectrophotometric determination of the DPPH, ABTS and FRAP antioxidant activities was obtained on lyophilized samples following the protocols described in detail by Formisano and collaborators [48]. For ABTS, 100 µL from a 1:10 dilution of sample material in 70% methanol was added to 1 mL of ABTS solution, and the 734 nm absorbance was recorded after 2.5 min. Similarly, DPPH results were obtained by adding 200 µL of the extract to 1 mL of DPPH solution; samples were incubated at ambient temperature for 10 min, and their 517 nm absorbance was recorded. Lastly, the ferric-reducing antioxidant power (FRAP) data were obtained by mixing 150 µL of the methanolic extract with 2.85 mL of FRAP solution. Samples were incubated for 4 min after which the 593 nm absorbance was read. All antioxidant activity results were expressed as mmol Trolox equivalents kg^−1^ DW.

### 4.7. Leaf Polyphenolic Contents

The leaf polyphenolic assay was performed analogously to the protocol followed by Kyriacou and collaborators [46]. Extraction was carried out on 100 mg of lyophilized leaf sample in 5 mL of a 60:40 *v*/*v* methanol/water solution. Phenolics separation was obtained via UHPLC system (Thermo Fisher Scientific, Waltham, MA, USA), equipped with 1.7 µm Biphenyl (100 × 2.1 mm) column (Phenomenex, Waltham, CA, USA). Mass spectrometry data were obtained via a Q Exactive Orbitrap LC-MS/MS (Thermo Fisher Scientific, Waltham, MA, USA). All polyphenolic data are expressed as mg kg^−1^ DW.

### 4.8. Statistical Analysis

Morpho-physiological and biochemical parameter data were analyzed with the SPSS 28 software package (IBM, Armonk, NY, USA) and are presented as mean ± standard error, *n* = 3. Data were first tested in order to meet the assumption of normality and homogeneity of variance using the Shapiro–Wilk and Levene tests, after which the mean effects were subjected to two-way (salinity level × biostimulant) ANOVA analysis. A *t* test was employed to compare the salinity mean effect, and Tukey’s HSD post hoc test was employed after a significant ANOVA test to separate both biostimulants mean effect and salinity × biostimulant interaction. All tests were deemed significant at *p* = 0.05. Principal component analysis (PCA) was performed on the studied parameters using the Minitab^®^ 18 software (Minitab LLC, State College, PA, USA), and the PCA biplot was obtained through the same software.

## 5. Conclusions

As we put two commercial PH biostimulants to the test against salt stress, we found an increase in yield of 8.9 and 4.6% by the Trainer and Vegamin biostimulants compared to the untreated control. We found that both biostimulants successfully managed to mitigate the salinity stress by modulating ion homeostasis parameters, which manifested in a decreased sodium accumulation and thus lower proline accumulation in the currently applied salt condition. This effect, coupled with the increase in photosynthesis parameters, has compounded the growth increases observed on the current genotype. In conclusion, we found confirmation that the effects of PH biostimulants on the physio-chemical and metabolic performance of lettuce plants are formulation-dependent, yet both the tested products provided increased plant growth in stress conditions, which can prove profitable in similar conditions. Deeper investigation on finer details of plant-stress response unveiled by this research in relation to the application of the PH biostimulants, such as increased root growth in salt stress conditions, root and shoot metabolic modulation and altered molecular pathways to fend off this particular stress, is warranted. A combined targeted metabolomic/transcriptomic approach may shed some more light on the inner workings of this product category.

## Figures and Tables

**Table 1:** Yield and yield parameters of lettuce plants as affected by salinity and biostimulant application.

Source of Variance	Leaf Number	Leaf Area	Shoot Fresh Weight	Leaf Dry Matter
	(no. Plant^−1^)	(cm^2^)	(g Plant^−1^)	(%)
**Salinity (S; mM NaCl)**				
0	34.7 ± 0.3	2357 ± 28 a	313.5 ± 3.3 a	5.29 ± 0.05 b
30	34.2 ± 0.4	2026 ± 37 b	240.5 ± 5.0 b	6.28 ± 0.03 a
*t*-test	ns	***	***	***
**Biostimulant (B)**				
Control	34.3 ± 0.5	2116 ± 72 b	265.1 ± 17.5 c	5.82 ± 0.22
Trainer	34.2 ± 0.1	2251 ± 60 a	288.7 ± 14.4 a	5.81 ± 0.24
Vegamin	34.8 ± 0.5	2207 ± 103 ab	277.2 ± 17.8 b	5.73 ± 0.22
	ns	*	***	ns
**S ** **× B**				
0 × Control	35.3 ± 0.6	2276 ± 12 ab	303.1 ± 4.7	5.34 ± 0.13
0 × Trainer	34.0 ± 0.2	2356 ± 50 a	320.7 ± 3.1	5.28 ± 0.11
0 × Vegamin	34.8 ± 0.6	2438 ± 10 a	316.7 ± 3.9	5.24 ± 0.02
30 × Control	33.4 ± 0.2	1957 ± 27 e	227.1 ± 8.1	6.29 ± 0.04
30 × Trainer	34.3 ± 0.2	2147 ± 69 bc	256.7 ± 1.1	6.33 ± 0.02
30 × Vegamin	34.9 ± 0.9	1976 ± 7.8 cd	237.7 ± 3.8	6.23 ± 0.08
	ns	*	ns	ns

All data are expressed as mean ± standard error, *n* = 3. ns, *, *** non-significant or significant at *p* ≤ 0.05 and 0.001, respectively. Nutrient solution dosage means were compared by *t* test. Biostimulant and S × B means were compared by two-way ANOVA. Different letters within each column indicate significant differences according to Tukey’s HSD (*p* = 0.05).

**Table 2:** Photosynthetic and biochemical parameters of lettuce plants as affected by salinity and biostimulant application.

Source of Variance	A_CO2_	g_s_	E	WUEi	Proline	MDA	H_2_O_2_
	(μmol CO_2_ m^−2^ s^−1^)	(mol H_2_O m^−2^ s^−1^)	(mol H_2_O m^−2^ s^−1^)	(μmol CO_2_ mol H_2_O^−1^)	(mM 100 g^−1^ FW)	(μM 100 g^−1^ FW)	(mM 100 g^−1^ FW)
**Salinity (S; mM NaCl)**							
0	17.73 ± 0.28	0.19 ± 0.00 a	3.96 ± 0.07 a	4.49 ± 0.12 b	19.4 ± 0.78 b	0.95 ± 0.04 b	4.66 ± 0.16 b
30	17.94 ± 0.74	0.16 ± 0.01 b	3.41 ± 0.14 b	5.33 ± 0.26 a	45.5 ± 2.28 a	1.05 ± 0.06 a	7.09 ± 0.22 a
*t*-test	ns	**	**	**	***	**	***
**Biostimulant (B)**							
Control	16.33 ± 0.44 b	0.16 ± 0.01 b	3.56 ± 0.23	4.66 ± 0.27 b	38.09 ± 7.26 a	1.17 ± 0.05 a	6.29 ± 0.66
Trainer	19.04 ± 0.38 a	0.17 ± 0.01 ab	3.65 ± 0.17	5.29 ± 0.33 a	29.29 ± 5.02 b	0.96 ± 0.04 b	5.66 ± 0.44
Vegamin	18.18 ± 0.43 a	0.19 ± 0.01 a	3.85 ± 0.13	4.66 ± 0.20 b	29.97 ± 5.35 b	0.87 ± 0.03 b	5.67 ± 0.62
	***	*	ns	*	***	***	ns
**S × B**							
0 × Control	17.13 ± 0.27 cd	0.18 ± 0.00	3.98 ± 0.06	4.31 ± 0.13	21.90 ± 0.94 c	1.08 ± 0.04 b	4.94 ± 0.28
0 × Trainer	18.57 ± 0.52 abc	0.19 ± 0.00	3.96 ± 0.12	4.71 ± 0.27	18.06 ± 0.38 c	0.88 ± 0.02 c	4.72 ± 0.21
0 × Vegamin	17.50 ± 0.18 bcd	0.19 ± 0.01	3.95 ± 0.19	4.45 ± 0.19	18.24 ± 1.22 c	0.89 ± 0.03 c	4.31 ± 0.27
30 × Control	15.53 ± 0.49 d	0.14 ± 0.00	3.15 ± 0.29	5.01 ± 0.47	54.29 ± 0.72 a	1.26 ± 0.03 a	7.64 ± 0.52
30 × Trainer	19.51 ± 0.49 a	0.16 ± 0.02	3.34 ± 0.17	5.87 ± 0.35	40.52 ± 0.11 b	1.04 ± 0.02 b	6.6 ± 0.19
30 × Vegamin	19.2 ± 0.01 ab	0.19 ± 0.02	3.74 ± 0.20	4.98 ± 0.33	41.70 ± 1.96 b	0.86 ± 0.05 c	7.04 ± 0.03
	**	Ns	ns	ns	**	*	ns

All data are expressed as mean ± standard error, *n* = 3. ns, *, **, *** non-significant or significant at *p* ≤ 0.05, 0.01 and 0.001, respectively. Nutrient solution dosage means were compared by *t* test. Biostimulant and S × B means were compared by two-way ANOVA. Different letters within each column indicate significant differences according to Tukey’s HSD (*p* = 0.05). A_CO2_, CO_2_ net assimilation rate, g_s_, stomatal conductance, E, transpiration, WUE_i_, intrinsic water use efficiency.

**Table 3:** Mineral contents of lettuce leaves as affected by salinity and biostimulant application.

Source of Variance	Total N	P	K	S	Ca	Mg	Na	Cl	Na/K Ratio
	(mg g^−1^ DW)	(mg g^−1^ DW)	(mg g^−1^ DW)	(mg g^−1^ DW)	(mg g^−1^ DW)	(mg g^−1^ DW)	(mg g^−1^ DW)	(mg g^−1^ DW)	
**Salinity (S; mM NaCl)**									
0	32.85 ± 0.32	4.52 ± 0.12	49.12 ± 1.72	0.76 ± 0.01 a	2.86 ± 0.09 a	1.89 ± 0.05 a	2.67 ± 0.25 b	8.74 ± 0.22 b	0.06 ± 0.01 b
30	32.84 ± 0.34	4.81 ± 0.13	53.59 ± 1.53	0.70 ± 0.02 b	2.20 ± 0.12 b	1.63 ± 0.04 b	9.92 ± 0.90 a	19.73 ± 0.87 a	0.18 ± 0.01 a
*t*-test	ns	ns	ns	*	**	***	***	***	***
**Biostimulant (B)**									
Control	32.2 ± 0.45	4.52 ± 0.15	50.72 ± 2.81	0.70 ± 0.02	2.33 ± 0.21 b	1.67 ± 0.04 b	7.91 ± 2.37 a	15.13 ± 2.72	0.15 ± 0.04 a
Trainer	33.3 ± 0.36	4.89 ± 0.17	52.80 ± 1.68	0.75 ± 0.02	2.79 ± 0.16 a	1.90 ± 0.07 a	5.47 ± 1.43 b	14.41 ± 2.89	0.10 ± 0.03 b
Vegamin	33.04 ± 0.25	4.59 ± 0.12	50.84 ± 2.02	0.74 ± 0.03	2.38 ± 0.17 b	1.68 ± 0.09 b	6.08 ± 1.31 b	14.06 ± 2.14	0.12 ± 0.03 ab
	ns	ns	ns	ns	*	**	**	ns	**
**S × B**									
0 × Control	32.13 ± 0.85	4.25 ± 0.16	44.50 ± 0.65 b	0.73 ± 0.02	2.79 ± 0.02	1.76 ± 0.03	2.62 ± 0.27 c	9.15 ± 0.34	0.06 ± 0.01 c
0 × Trainer	33.27 ± 0.23	4.67 ± 0.21	51.57 ± 2.60 ab	0.78 ± 0.02	3.04 ± 0.11	2.03 ± 0.05	2.40 ± 0.63 c	8.26 ± 0.28	0.05 ± 0.01 c
0 × Vegamin	33.13 ± 0.26	4.70 ± 0.12	52.38 ± 3.15 ab	0.78 ± 0.03	2.68 ± 0.34	1.89 ± 0.07	3.14 ± 0.24 c	8.84 ± 0.39	0.06 ± 0.01 c
30 × Control	32.27 ± 0.52	4.78 ± 0.10	56.94 ± 0.47 a	0.66 ± 0.03	1.87 ± 0.11	1.58 ± 0.03	13.19 ± 0.46 a	21.11 ± 1.05	0.23 ± 0.01 a
30 × Trainer	33.32 ± 0.77	5.11 ± 0.24	54.03 ± 2.43 ab	0.72 ± 0.03	2.53 ± 0.23	1.76 ± 0.07	8.54 ± 0.66 b	20.55 ± 2.01	0.16 ± 0.01 b
30 × Vegamin	32.95 ± 0.47	4.53 ± 0.19	49.81 ± 3.01 ab	0.71 ± 0.03	2.19 ± 0.06	1.54 ± 0.03	8.04 ± 0.96 b	17.53 ± 0.37	0.16 ± 0.01 b
	ns	ns	*	ns	ns	ns	**	ns	**

All data are expressed as mean ± standard error, *n* = 3. ns, *, **, *** non-significant or significant at *p* ≤ 0.05, 0.01 and 0.001, respectively. Nutrient solution dosage means were compared by *t* test. Biostimulant and S × B means were compared by two-way ANOVA. Different letters within each column indicate significant differences according to Tukey’s HSD (*p* = 0.05).

**Table 4:** Auxiliary pigment content and antioxidant activity of lettuce leaves as affected by salinity and biostimulant application.

Source of Variance	Lutein	β-carotene	DPPH	ABTS	FRAP
	(mg kg^−1^ DW)	(mg kg^−1^ DW)	(mmol Trolox kg^−1^ DW)	(mmol Trolox kg^−1^ DW)	(mmol Trolox kg^−1^ DW)
**Salinity (S; mM NaCl)**					
0	427.8 ± 11.2 b	219.5 ± 8.1 b	33.71 ± 1.58 b	43.60 ± 2.33 b	41.05 ± 2.80 b
30	595.6 ± 39.3 a	286.0 ± 11.3 a	36.85 ± 1.22 a	48.62 ± 3.68 a	46.05 ± 2.95 a
*t*-test	***	***	***	*	***
**Biostimulant (B)**					
Control	474.4 ± 19.7 b	254.3 ± 12.7 ab	30.23 ± 1.18 c	35.91 ± 1.31 c	33.70 ± 1.27 c
Trainer	585.9 ± 72.1 a	278.4 ± 20.5 a	39.49 ± 0.63 a	54.84 ± 2.70 a	53.31 ± 1.65 a
Vegamin	474.7 ± 31.4 b	225.6 ± 16.3 b	36.13 ± 0.71 b	47.57 ± 1.94 b	43.64 ± 0.87 b
	**	**	***	***	***
**S × B**					
0 × Control	436.5 ± 17.3 b	227.6 ± 7.7	27.83 ± 0.57	34.69 ± 0.69	30.89 ± 0.18
0 × Trainer	428.6 ± 27.0 b	234.9 ± 14.6	38.30 ± 0.36	49.77 ± 0.75	50.12 ± 0.54
0 × Vegamin	418.3 ± 20.1 b	196.1 ± 9.8	35.00 ± 0.99	46.32 ± 1.24	42.14 ± 0.91
30 × Control	512.3 ± 14.1 b	281.0 ± 6.2	32.63 ± 0.94	37.13 ± 2.58	36.50 ± 0.42
30 × Trainer	743.2 ± 22.5 a	321.8 ± 2.4	40.68 ± 0.67	59.91 ± 3.17	56.50 ± 1.78
30 × Vegamin	531.1 ± 36.8 b	255.2 ± 19.2	37.25 ± 0.56	48.82 ± 3.97	45.14 ± 0.83
	**	ns	ns	ns	ns

All data are expressed as mean ± standard error, *n* = 3. ns, *, **, *** non-significant or significant at *p* ≤ 0.05, 0.01 and 0.001, respectively. Nutrient solution dosage means were compared by *t* test. Biostimulant and S × B means were compared by two-way ANOVA. Different letters within each column indicate significant differences according to Tukey’s HSD (*p* = 0.05).

**Table 5:** The modulation of the leaf content of phenolic acids as affected by salinity and biostimulant application.

Source of Variance	Chlorogenic Acid	Coumaroyl-diglucoside	Disinapoylgentobiose	Ferulic Acid	Synapoyl-hexose	Total Phenolic Acids
	(mg kg^−1^ DW)	(mg kg^−1^ DW)	(mg kg^−1^ DW)	(mg kg^−1^ DW)	(mg kg^−1^ DW)	(mg kg^−1^ DW)
**Salinity (S; mM NaCl)**						
0	1278 ± 86 b	1.51 ± 0.1 b	0.39 ± 0.02 b	37.67 ± 4.18	46.54 ± 1.79	1364 ± 89 b
30	1439 ± 77 a	1.88 ± 0.16 a	0.44 ± 0.03 a	39.87 ± 2.87	48.98 ± 3.82	1530 ± 80 a
*t*-test	*	***	**	ns	ns	*
**Biostimulant (B)**						
Control	1078 ± 57 b	1.25 ± 0.05 c	0.32 ± 0.02 c	33.59 ± 2.23	39.63 ± 2.63 c	1153 ± 57 b
Trainer	1551 ± 65 a	2.12 ± 0.15 a	0.48 ± 0.02 a	43.7 ± 4.87	56.17 ± 2.6 a	1654 ± 68 a
Vegamin	1447 ± 61 a	1.72 ± 0.09 b	0.43 ± 0.00 b	39.02 ± 4.84	47.48 ± 1.56 b	1536 ± 58 a
	***	***	***	ns	***	***
**S × B**						
0 × Control	953 ± 5.9	1.16 ± 0.05	0.30 ± 0.00	28.69 ± 0.65	43.35 ± 4.32 bc	1026 ± 3.6
0 × Trainer	1472 ± 53	1.81 ± 0.07	0.43 ± 0.00	45.53 ± 9.98	50.77 ± 1.98 ab	1571 ± 63.2
0 × Vegamin	1410 ± 72	1.57 ± 0.11	0.42 ± 0.00	38.78 ± 6.11	45.5 ± 1.04 bc	1497 ± 69.4
30 × Control	1203 ± 19	1.34 ± 0.05	0.34 ± 0.03	38.5 ± 0.63	35.91 ± 1.4 c	1280 ± 20.7
30 × Trainer	1631 ± 110	2.43 ± 0.06	0.53 ± 0.01	41.86 ± 3.95	61.56 ± 0.89 a	1737 ± 111
30 × Vegamin	1483 ± 111	1.86 ± 0.10	0.44 ± 0.00	39.26 ± 8.93	49.46 ± 2.69 b	1574 ± 102
	ns	ns	ns	ns	*	ns

All data are expressed as mean ± standard error, *n* = 3. ns, *, **, *** non-significant or significant at *p* ≤ 0.05, 0.01 and 0.001, respectively. Nutrient solution dosage means were compared by *t* test. Biostimulant and S × B means were compared by two-way ANOVA. Different letters within each column indicate significant differences according to Tukey’s HSD (*p* = 0.05).

**Table 6:** The modulation of the leaf content of flavonoids as affected by salinity and biostimulant application.

Source of Variance	Isorhamnetin 3-rutinoside	Kaempferol 3,7-diglucoside	Kaempferol 3-glucoside	Quercetin 3-glucoside	Rutin	Total Flavonoids
	(mg kg^−1^ DW)	(mg kg^−1^ DW)	(mg kg^−1^ DW)	(mg kg^−1^ DW)	(mg kg^−1^ DW)	(mg kg^−1^ DW)
**Salt (S; mM NaCl)**						
0	0.37 ± 0.05 b	0.33 ± 0.02 b	1.21 ± 0.10 b	10.31 ± 1.27 b	0.55 ± 0.1 b	12.77 ± 1.49 b
30	0.55 ± 0.04 a	0.48 ± 0.04 a	1.39 ± 0.09 a	12.10 ± 0.93 a	0.72 ± 0.12 a	15.25 ± 1.12 a
*t*-test	***	***	***	***	***	***
**Biostimulant (B)**						
Control	0.29 ± 0.06 c	0.52 ± 0.05 a	0.95 ± 0.06 c	7.44 ± 0.72 c	0.20 ± 0.03 b	9.41 ± 0.9 c
Trainer	0.58 ± 0.04 a	0.38 ± 0.03 b	1.56 ± 0.05 a	14.88 ± 0.35 a	0.90 ± 0.05 a	18.3 ± 0.42 a
Vegamin	0.50 ± 0.03 b	0.32 ± 0.04 b	1.40 ± 0.03 b	11.3 ± 0.50 b	0.81 ± 0.06 a	14.33 ± 0.58 b
	***	***	***	***	***	***
**S × B**						
0 × Control	0.17 ± 0.04 d	0.41 ± 0.02	0.81 ± 0.03 d	5.91 ± 0.47 d	0.15 ± 0.01	7.46 ± 0.48 d
0 × Trainer	0.49 ± 0.01 bc	0.32 ± 0.01	1.45 ± 0.02 b	14.57 ± 0.39 a	0.79 ± 0.04	17.62 ± 0.37 a
0 × Vegamin	0.45 ± 0.02 c	0.25 ± 0.01	1.38 ± 0.04 b	10.46 ± 0.51 bc	0.70 ± 0.02	13.24 ± 0.44 c
30 × Control	0.42 ± 0.01 c	0.63 ± 0.02	1.09 ± 0.02 c	8.97 ± 0.09 c	0.25 ± 0.04	11.36 ± 0.14 c
30 × Trainer	0.67 ± 0.02 a	0.44 ± 0.01	1.67 ± 0.02 a	15.18 ± 0.6 a	1.01 ± 0.01	18.97 ± 0.56 a
30 × Vegamin	0.56 ± 0.02 b	0.39 ± 0.04	1.42 ± 0.04 b	12.14 ± 0.55 b	0.91 ± 0.09	15.42 ± 0.56 b
	*	ns	**	*	ns	*

All data are expressed as mean ± standard error, *n* = 3. ns, *, **, *** non-significant or significant at *p* ≤ 0.05, 0.01 and 0.001, respectively. Nutrient solution dosage means were compared by *t* test. Biostimulant and S × B means were compared by two-way ANOVA. Different letters within each column indicate significant differences according to Tukey’s HSD (*p* = 0.05).

## Data Availability

The datasets generated for this study are available on request to the corresponding author.
